# Asymptomatic *Plasmodium falciparum* infection in children is associated with increased auto-antibody production, high IL-10 plasma levels and antibodies to merozoite surface protein 3

**DOI:** 10.1186/s12936-015-0658-7

**Published:** 2015-04-16

**Authors:** Vincent Guiyedi, Christophe Bécavin, Fabien Herbert, Julian Gray, Pierre-André Cazenave, Maryvonne Kombila, Andrea Crisanti, Constantin Fesel, Sylviane Pied

**Affiliations:** CIIL-Centre for Infection and Immunity of Lille, INSERM U1019 - CNRS UMR 8204, Lille University, Institut Pasteur de Lille, 1, rue du Professeur Calmette, Cedex 59019 Lille, France; Département de Parasitologie-Mycologie-Médecine Tropicale, Faculté de Médecine de Libreville, Université des Sciences de la Santé, Owendo, Gabon; Département de Biologie Cellulaire et Infection, Institut Pasteur, Unité des Interactions Bactéries-Cellules, F-75015 Paris, France; Department of Biological Sciences, London Imperial College, London, UK; Instituto Gulbenkian de Ciência, Oeiras, Portugal

**Keywords:** Auto-antibody, Antibody response, *Plasmodium falciparum*, Malaria, Cytokines

## Abstract

**Background:**

Mechanisms of acquired protection to malaria in asymptomatic *Plasmodium falciparum* carriers are only partially understood. Among them, the role plays by the self-reactive antibodies has not been clarified yet. In this study, the relationship between repertoires of circulating self-reactive and parasite-specific immunoglobulin G (IgG), their correlation with cytokine levels, and their association with protection against malaria was investigated in asymptomatic *Plasmodium falciparum*-infected Gabonese children.

**Methods:**

The diversity of *P. falciparum*-specific antibody repertoire was analysed using a protein micro-array immunoassay, the total auto-antibody repertoire by quantitative immunoblotting and circulating cytokine levels were measured by ELISA in endemic controls (EC) and *P. falciparum*-infected children from Gabon with asymptomatic (AM) or mild malaria (MM). The association of self- and parasite-specific antibody repertoires with circulating cytokines was evaluated using single linkage hierarchical clustering, Kruskal – Wallis tests and Spearman’s rank correlation.

**Results:**

Children with AM exhibited an IgG response to merozoite surface protein 3 (MSP3) but not to MSP1-19, although their levels of total *P. falciparum*-specific IgG were similar to those in the MM group. Moreover, the asymptomatic children had increased levels of autoantibodies recognising brain antigens. In addition, a correlation between IL-10 levels and parasite load was found in AM and MM children. These two groups also exhibited significant correlations between plasma levels of IL-10 and IFN-γ with age and with total plasma IgG levels. IL-10 and IFN-γ levels were also associated with auto-antibody responses in AM.

**Conclusions:**

Altogether, these results indicate that a self-reactive polyclonal response associated with increased IgG to MSP3 and high plasma levels of IL-10 and IFN-γ may contribute to protective immune mechanisms triggered in asymptomatic *P. falciparum* infection in Gabonese children.

**Electronic supplementary material:**

The online version of this article (doi:10.1186/s12936-015-0658-7) contains supplementary material, which is available to authorized users.

## Background

*Plasmodium falciparum* infection can lead to asymptomatic malaria (AM), mild malaria (MM) or severe malaria (SM) [1]. Despite considerable research, the mechanisms of naturally acquired immunity to malaria are only partially understood. Natural protection against malaria is acquired after years of continuous exposure to infectious mosquito bites [2], and such clinical immunity is multifactorial. Besides a cellular response, antibody-mediated effector mechanisms are implicated in protective immunity [3-5]. However, this protection is against severe disease, not re-infection. Clearly, long-term immunity to malaria is characterized by the ability to reduce, but not eliminate, the parasite load and, therefore, to better tolerate disease − the ‘premunition’ defined by Sergent [2]. There is still a need to identify immune response components of clinical immunity.

Malaria is associated with hypergammaglobulinemia and the production of self-reactive antibodies that recognize self-antigens, such as phospholipid, cardiolipin, ssDNA, dsDNA, and rheumatoid factor [6-9], although they may also recognize parasite antigens. However, whether these self-reactive antibodies play a role in protection against parasite infection or severe disease is unclear. It is thus of critical importance to quantitatively study the range of antibody reactivities to understand the complexity of the humoral immune response to *P. falciparum*.

Several mechanisms may contribute to auto-antibody production in malaria. These include polyclonal B cell activation by parasitic mitogens [8], stimulation of B cells by molecular mimicry [10,11], dysregulation of B cell functions [12], and stimulation of B cells involved in auto-antibody production [13]. An integrated approach has been implemented in an endemic area of Libreville in Gabon to study the diversity of auto-antibody and parasite-specific repertoires in *P. falciparum*-infected children with either AM or MM and in healthy children (endemic control (EC)). The global analysis of antibody repertoires was done using a microarray immunoassay [14] and quantitative auto-antibody immunoblots [15-18] coupled to bio-informatics and statistics. For insights into the regulatory factors involved, the association of antibody repertoires with circulating cytokine patterns was also investigated in AM or MM and EC from an endemic area of Libreville, Gabon.

Results clearly show that IgG autoreactivity was greater in AM than in MM children. In addition, the auto-antibody response was correlated significantly with the presence of anti-merozoite surface protein antigen 3 (MSP3) IgG and high levels of IL-10 in the AM group. By contrast, anti-MSP1 IgG was predominant in MM patients. Interestingly, IL-10 levels were also associated with parasite load in AM and MM groups.

Altogether, these results strongly support a protective role for auto-antibodies triggered by the parasite which correlate with plasma IL-10 levels and with anti-MSP2 and anti-MSP3 IgG levels in clinical immunity observed in asymptomatic *P. falciparum* carriers.

## Methods

### Study population

The ethics committee of the Gabon Health Office approved this study. Between 1996 and 2000, 97 children (age range two months to six years) living in Libreville Hospital Centre (Gabon) were included in the study after obtaining parental informed consent. All patients presenting diseases other than malaria were excluded from the study. Malaria patients were categorized on the basis of World Health Organization (WHO) guidelines for the definition of uncomplicated malaria. Mild malaria (MM) presented fever with positive thin blood smears. Individuals with positive *P. falciparum* thin blood smears and no clinical symptoms were included in the asymptomatic malaria (AM) group and were defined as AM carriers and children with no clinical symptoms and a negative thin blood smears were enrolled in the endemic control (EC) group. All AM and EC children were from the same area of Libreville city. MM were recruited at Owendo Pediatric Hospital (OPH) and Libreville Hospital Centre. AM and EC children were examined daily for clinical symptoms. Parasitaemia were determined on days 0 (day of hospitalization), 7 and 30. Oral amodiaquine (25 mg/kg) was administered for three days from day-0 to MM patients and at day 30 to AM carriers. No patient death occurred during the recruitment period.

### Blood sample collection and parasite assessment

Venous blood was collected in EDTA on days 0 (before treatment), 7 and 30. Plasma was separated and stored at −80°C until use. Parasitaemia (expressed as the percentage of infected erythrocytes) was determined by microscopic examination of Giemsa-stained thin blood smears.

### Cytokine assays

Plasmatic levels of IFN-γ, TNF and IL-10 were measured in EC, AM and MM at day 0, 7 and 30 using a sandwich-type ELISA (Kit OptEIA set, Pharmingen, BD Bioscience, France) according to the manufacturer’s recommendations.

### Determination of plasmatic levels of total immunoglobulin G

The quantification of total IgG in children plasma from all groups was done by ELISA. At day 0, 7 and 30 after their inclusion in the study. Briefly, 96 microwell plates (reacti-bind 96 EIA Plate 100/PKG, Pierce) were coated with 5 μg/ml of purified sheep polyclonal anti-human IgG (Sigma-Aldrich, France) followed by overnight incubation at 4°C. After blockage with 1% gelatine and washing with 1% gelatin-PBS, serial dilutions of plasma samples were incubated overnight at 4°C. Bound IgG was detected using a peroxidase-conjugated polyclonal anti-human-IgG (The Binding Site, Birmingham UK). Binding was revealed using 0.5 mg/ml O-phenylenediamine (OPD) substrate (Sigma-Aldrich, France) and 0.03% H_2_O_2_ in 0.05 M phosphate-citrate buffer, pH5 and the product was quantified from the optical density (OD) at 450 nm. The mean of OD value was fitted into the sigmoidal standard curve using a specific ELISA programme running in Igor version 3.16 (Wavemetrics, Lake Oswego, OR).

### Total *Plasmodium falciparum*-specific immunoglobulin G quantification

Total *P. falciparum* specific plasmatic IgGs were quantified using ELISA. Briefly, ninety-six-well plates (Reacti-Bind 96 EIA Plat Corn Not, Pierce) were coated overnight at 4°C with 5 μg/ml of *P. falciparum* 3D7 schizont-stage protein extract. Plates were blocked with 1% gelatine. One-hundred ml of 1:100 dilution of patient plasma was added to each well in duplicate, incubated for one hour at 37°C, and developed using anti-human IgG-HRP and OPD (Sigma-Aldrich, France). Ten per cent sodium dodecyl sulphate was added after 30 min and the OD measured at 450 nm. Concentration of *P. falciparum*-specific IgG in the samples was calculated from a standard curve using 0.001 − 2 mg/ml of purified human IgG. For every sample tested a cut-off value was estimated using a pool of plasma from children living in Europe who had never been exposed to *P. falciparum* infection.

### Determination of *Plasmodium falciparum*-specific antibody repertoire

The plasma parasite-specific antibodies were profiled using a protein micro-array immuno-assay [14]. Each array contained 20 recombinant antigens derived from leading blood-stage vaccine candidates, including antigenic variants of MSP1, MSP2 and MSP3, apical membrane antigen 1 (AMA1), *P. falciparum* erythrocyte membrane protein 1 (PfEMP1), and circumsporozoite protein epitope NANP-4. Arrays were incubated with 100 μl of 1:100 dilution of plasma for 15 min at 37°C and washed for 5 min in PBS-Tween. Arrays were then incubated with Alexa Fluor 546-labelled anti-human IgG monoclonal antibody (1:1,000; Jackson Immunoresearch Europe, UK) for 20 min. Alexa 555 diluted 1:1 in PBS was used as signal control. Ten mg/ml bovine serum albumin was used as a negative control. Anti-human IgG (Sigma, France) was used as carry-over control. Arrays were washed twice in PBS-Tween, dried under nitrogen, and scanned with a GMS 428 scanner (Affymetrix, Santa Clara, CA, USA). Acquired images were analysed with the GenePix image analysis software (version 3.0.6.89, Axon Instruments, CA, USA). The mean intensity of duplicate spots was analysed using the linear range of the individual slide standard micro-array [14].

### Brain antigen preparation, gel electrophoresis, immunoblotting, and data treatment

The PANAMA-Blot method was used to determine the antibody repertoire to brain antigens as previously described [15,16]. PANAMA-Blot is a powerful approach allowing the analysis of the self-reactive antibodies repertoire against a large panel of antigens. Briefly, proteins from normal human brain, from a volunteer donor after death, were separated on 10% SDS-PAGE at 25 mA, and were transferred onto nitrocellulose membranes (Schleicher & Schull, Dassel, Germany) by semi-dry electrotransfer (Pasteur Institute, Paris) for 1 h at 0.8 mA/cm2. Membranes were blocked by incubation overnight at room temperature with 0.2% Tween 20 in PBS (PBST), and were then incubated with plasma samples diluted 1:20 in PBST (non-adjusted assay), or at a total IgG concentration of 200 μg/ml (adjusted assay), in a Cassette Miniblot System (Immunetics, MA, USA). Membranes were incubated for 4 h at room temperature with gentle shaking. Bound IgG were detected with rabbit anti-human IgG–alkaline phosphatase (Sigma-Aldrich, France) and nitroblue tetrazolium/5-bromo-4-chloro-3-indolyl-phosphate (Promega, France) incubated for 90 minutes at room temperature. Immunoreactivity was detected with NBT/BCPI (nitroblue tetrazolium/5-bromo-4-chloro-3-indolyl-phosphate) and the quantitative signals were normalized using the IGOR 3.16 software (Wavemetrics, Lake Oswego, OR, USA). A standard plasma sample (a mixture of diverse plasma samples from Gabonese individuals diluted 1:20) was applied in duplicate for intensity adjustment.

### Statistical analysis

Immunoblot data were analysed by multivariate statistical methods, using IGOR software (Wavemetrics, Lake Oswego, OR, USA). After baseline subtraction, the standard migration scale was divided into sections around individual peaks of immunoreactivity. Section-wise absorbance values were subjected to principal component analysis (PCA). The first component (PCA-1) was the one-dimensional fitting vector that accounted for the largest proportion of variance and factor 2 (PCA-2) was the second-best orthogonal one-dimensional vector fitting the data. Quantitative comparisons between groups were done either with the Mann–Whitney (between two groups) or Kruskal-Wallis tests (>two groups). Qualitative association was tested by Pearson’s c^2^ test. The association between continuous quantitative parameters was assessed by Spearman’s rank correlation. The trend over time was tested by the paired *t*-test; p-values <0.05 were considered significant. Linear discriminant analysis was performed on log-transformed data, and samples with incomplete information were rejected. The significance of contributing parameters was calculated by backward elimination, using partial F tests on variances explained by models with or without the respective parameters. The significance criterion was a Bonferroni-corrected p-value <0.05. Single linkage hierarchical clustering of the antibody response against the 20 *P. falciparum* antigens tested in the different groups was performed using the hclust function in R software. For the heatmaps, fold change of the antibody response and was calculated using the formula Fc = μ1/μ2 for μ1 > μ2 and Fc = −μ2/μ1 for μ1 < μ2, where μ1 is the mean of the first group and μ2 the mean of the second group compared. The same methods were used for brain self-reactive IgG responses clustering and heatmaps.

## Results

### Distribution of total and *Plasmodium falciparum*-specific IgG in EC, AM and MM groups

**Figure 1:**
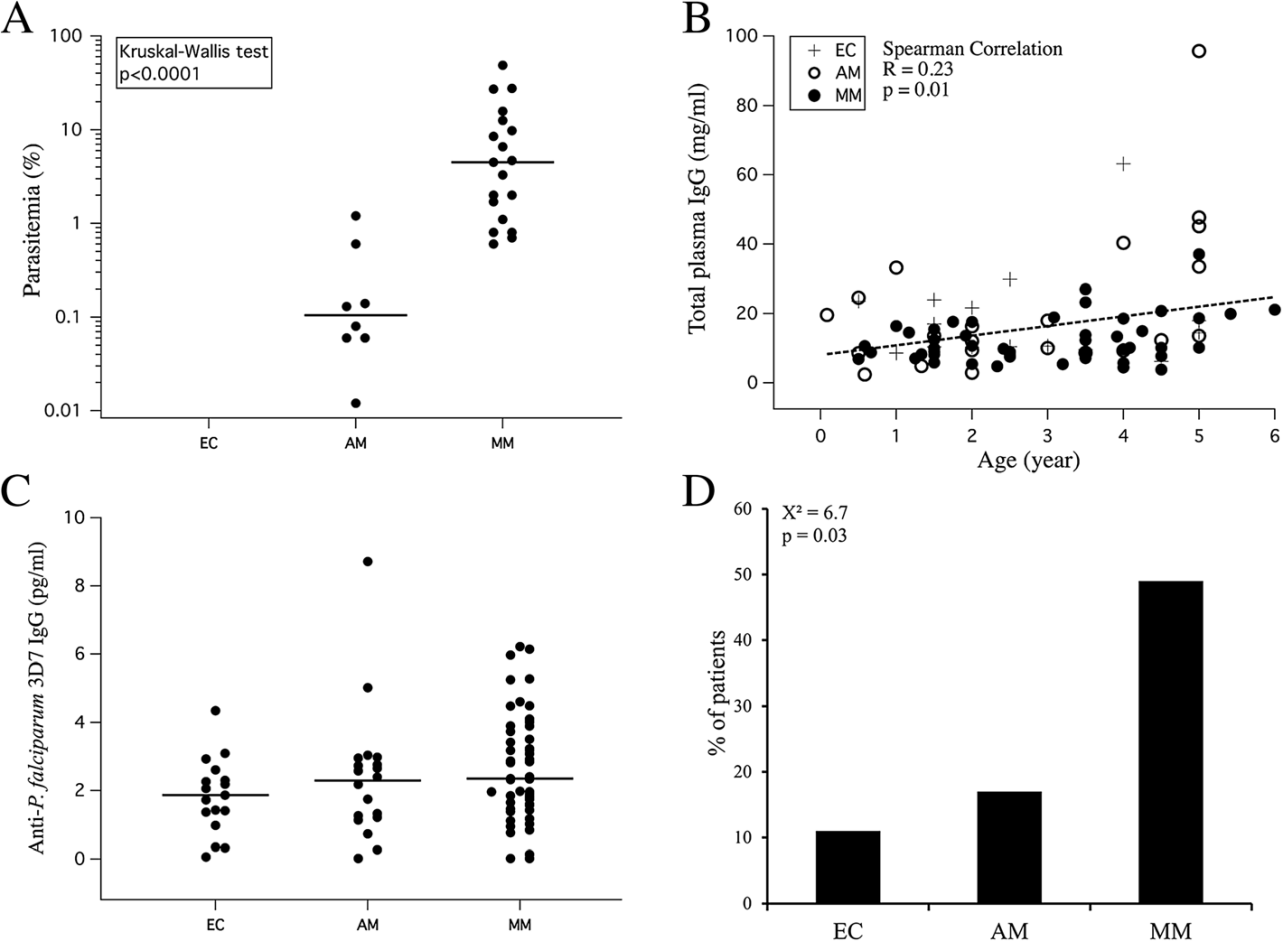
Epidemiological and serological characteristics of the study population. **(A)** Parasite loads (percentage of *P. falciparum*-infected red blood cells) in the EC, AM and MM groups. **(B)** Correlation between total IgGs and age of the children in EC, AM and MM groups. **(C)** Levels of IgG recognizing total *P. falciparum* antigens in the EC, AM and MM groups. **(D)** Proportion of patients with >3 pg/ml of *P. falciparum*-specific IgG in the respective clinical groups.

Three groups of patients were studied: 18 in EC, 22 in AM, and 57 in MM. Sex distribution and mean age were not significantly different in the three groups (Table 1). The median parasitaemia was significantly lower in AM than in MM (0.11% (range 0.01 − 1.2) *versus* 4.5% (range 0.6 − 48.6); p <0.001), (Figure 1A). Total IgG levels at day 0 (hospitalisation day) before treatment were not significantly different in the three groups (Table 1). There was no correlation between parasite load and total IgG levels. However, a significant increase with age of total IgG concentrations was observed at day 0 (Spearman R = 0.23, p = 0.01), regardless of group (Figure 1B). Levels of *P. falciparum*-specific IgG in the three groups were also statistically indistinguishable (Figure 1C). It is noteworthy that 49% of patients in MM group had highest levels of anti-*P. falciparum* IgG at day 0 (levels >3 pg/ml), compared to 17% in AM and 11% in EC (overall c2 = 6.7, p = 0.03), (Figure 1D). There was no correlation between *P. falciparum*-specific IgG levels and parasite loads.

**Table 1:** **General characteristics of patients in the different clinical groups**

**Group**	**Patient no. (%)**	**Mean age (range)**	**Sex ratio (M/F)**	**Mortality**	**Total IgG mean in mg/ml (SD)**
EC	18 (18.56)	2.6 (0.5 – 5)	10/8	0	18.5 (13.0)
AM	22 (22.68)	2.8 (0.1 – 5)	12/10	0	23.8 (22.8)
MM	57 (58.76)	3 (0.5 – 6)	20/30*	0	13.5 (7.6)
Total	97 (100)	2.8 (0.1 – 6)	42/48	0	17.02 (14.3)

EC: uninfected controls, AM: asymptomatic *P. falciparum-*infected patients, MM: Mild Malaria, SD: standard deviation.

*Not determined for 7 patients.

Levels of total Immunoglobulin G were quantified in all groups on day of inclusion in the study and before treatment for MM patients (corresponding to day 0).

### Serological evaluation of the repertoire of *Plasmodium falciparum*-specific IgG in AM and MM patients

**Figure 2:**
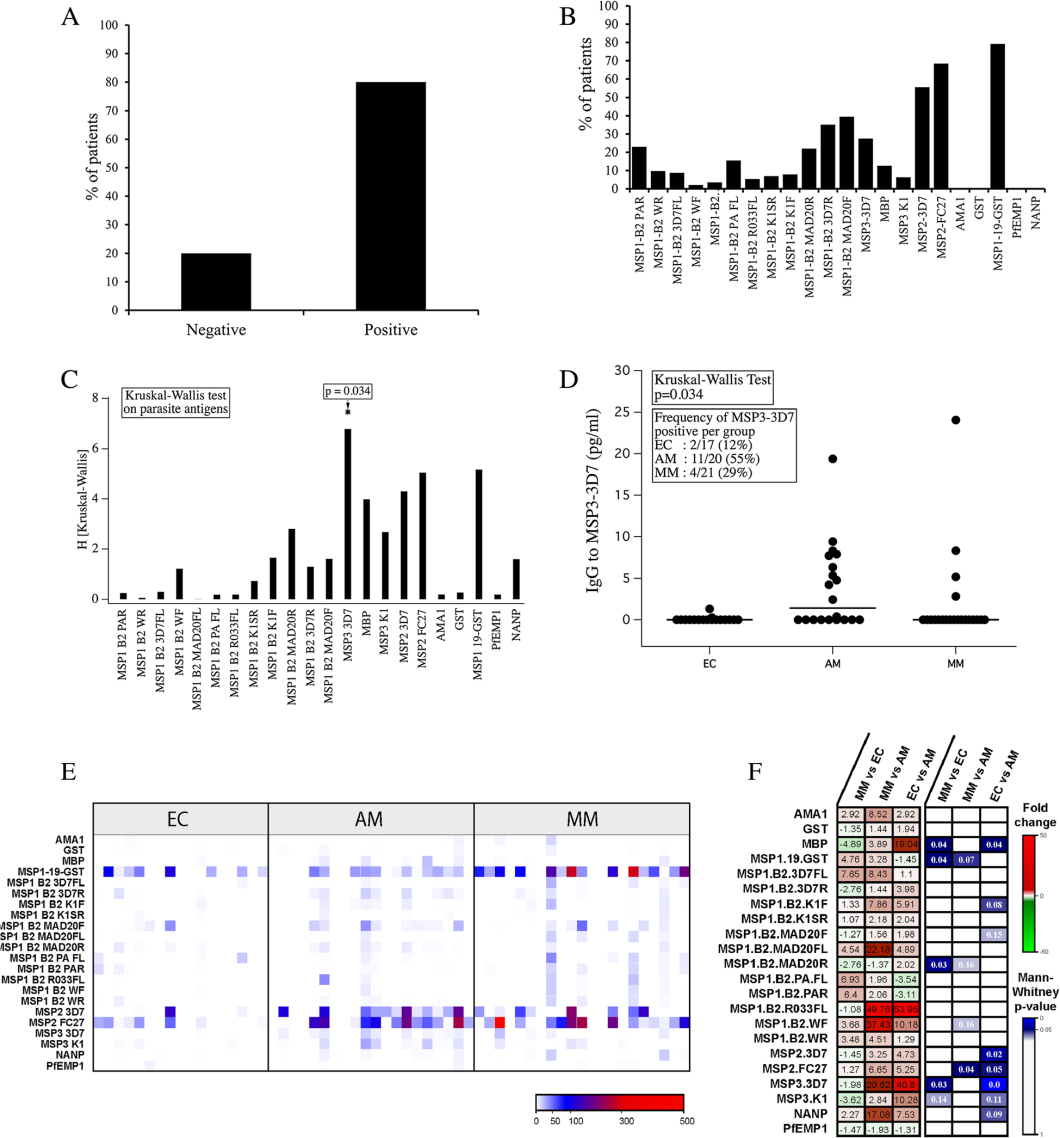
Repertoire of *Plasmodium falciparum* antigenic diversity recognized by infected children in Gabon. **(A)** Percentage of individuals in the study population that recognized at least one *P. falciparum* antigen. **(B)** Antibody patterns and percentage of positive patients for each of the 20 *P. falciparum*-specific antigens tested in the total study population. **(C)** Kruskal-Wallis analysis of the antibody responses against the 20 parasite antigens in all groups. **(D)** Plasma levels (pg/mL) of anti-*P. falciparum* MSP3-3D7 antibodies in EC, AM and MM. **(E)** Single linkage hierarchical clustering of the antibody response against the 20 *P. falciparum* antigens tested in the different groups. **(F)** Heat map representing fold change of the antibody response and Mann–Whitney p-value comparing the different groups: AM *vs* MM, AM *vs* EC, and EC *vs* MM. Antigens: MSP1 block 2 PA repeats; MSP1 block 2 3D7 Wellcome repeats; MSP1 block 2 3D7 full length; MSP1 block 2 3D7 Wellcome full length; MSP1 block 2 MAD20 full length; MSP1 block 2 RO33 full length; MSP1 block 2 K1 super repeats; MSP1 block 2 K1 flanking; MSP1 block 2 MAD20 repeats; MSP1 block 2 3D7 repeats; MSP3 3D7; MBP; MSP3 K1; MSP2 3D7; MSP2 FC27; AMA-1; GST; MSP1-19 GST; PfEMP1; NANP.

The repertoires of parasite antigens recognized by EC, AM and MM were compared using a protein micro-array consisting of 20 antigens, including 13 variants of MSP1, MSP2 and MSP3 from *P. falciparum* 3D7 and FC27 clones, AMA1, PfEMP1, and NANP-4. The results showed that 80% of the studied population, including EC, recognized at least one of the *P. falciparum* antigens tested while 20% had any reactivity (Figure 2A). In addition, 75% of MM recognized the highest number of *P. falciparum* antigens when compared to 15% in AM and 10% in EC. When grouped together, 60% of the studied population had IgG to MSP2, 75% IgG to MSP1-19 IgG and less than 40% had IgG to at least one the other tested antigens (Figure 2B). It is worthy to note that no reactivity was observed against AMA1, PfEMP1, GST and NANP-4. The most significant difference between the groups was observed by Kruskal-Wallis for MSP3-3D7 (Figure 2C; p = 0.034). Plasma levels (pg/mL) of circulating anti-MSP3-3D7 IgG was higher in the AM group than in the MM and EC groups (Figure 2D; p = 0.03). It is also noteworthy that 55% of AM compared to 12% EN and 19% MM produced IgG anti-MSP3-3D7. Conversely, the level of anti-MSP1-19 antibody was significantly higher in the MM than in AM and EC [see Additional file 1]. In the same patient group, IgG to MSP2-3D7 or MSP2-FC27 detected in some MM patients on day 0 persisted with the same intensity until day 30, even after treatment [see Additional file 2A]. However, for some of the antigens, such as MSP1-19, reactivity increased between days 0 and 7 or between days 0 and 30 [see Additional file 2B].

Single linkage hierarchical clustering was then applied to compare the repertoire of antigenic specificities recognized by circulating IgG of *P. falciparum* infected individuals at day 0 before the start of drug therapy as well as for EC. Interestingly, AM and MM groups typically recognized MSP2 and children in AM group showed a predominant IgG response against two MSP2 variants (FC 27 and MSP2-3D7) compared to the EC group, whereas IgG to MSP1-B2 PAR distinguished MM from AM (Figure 2E). By contrast, the EC group showed a predominant response to MSP1-19 GST (Figure 2E) and to anti- Myelin Basic Protein (MBP) used as an irrelevant antigen (Figure 2F).

The differences in fold change of *P. falciparum* specific IgG response between groups were also assessed using an unpaired *t*-test. AM individuals produced significantly greater anti-MSP3 IgG than EC individuals, whereas anti-MSP1 IgG was predominant in MM patients (Figure 2F). These data show that MSP1 from *P. falciparum* 3D7 strain are recognized by the IgG of patients with disease (MM), whereas MSP3 and MSP2 antigens are predominantly recognized by IgG of AM carriers.

### Profiles of self-reactive IgG to brain antigens in EC, AM and MM groups

**Figure 3:**
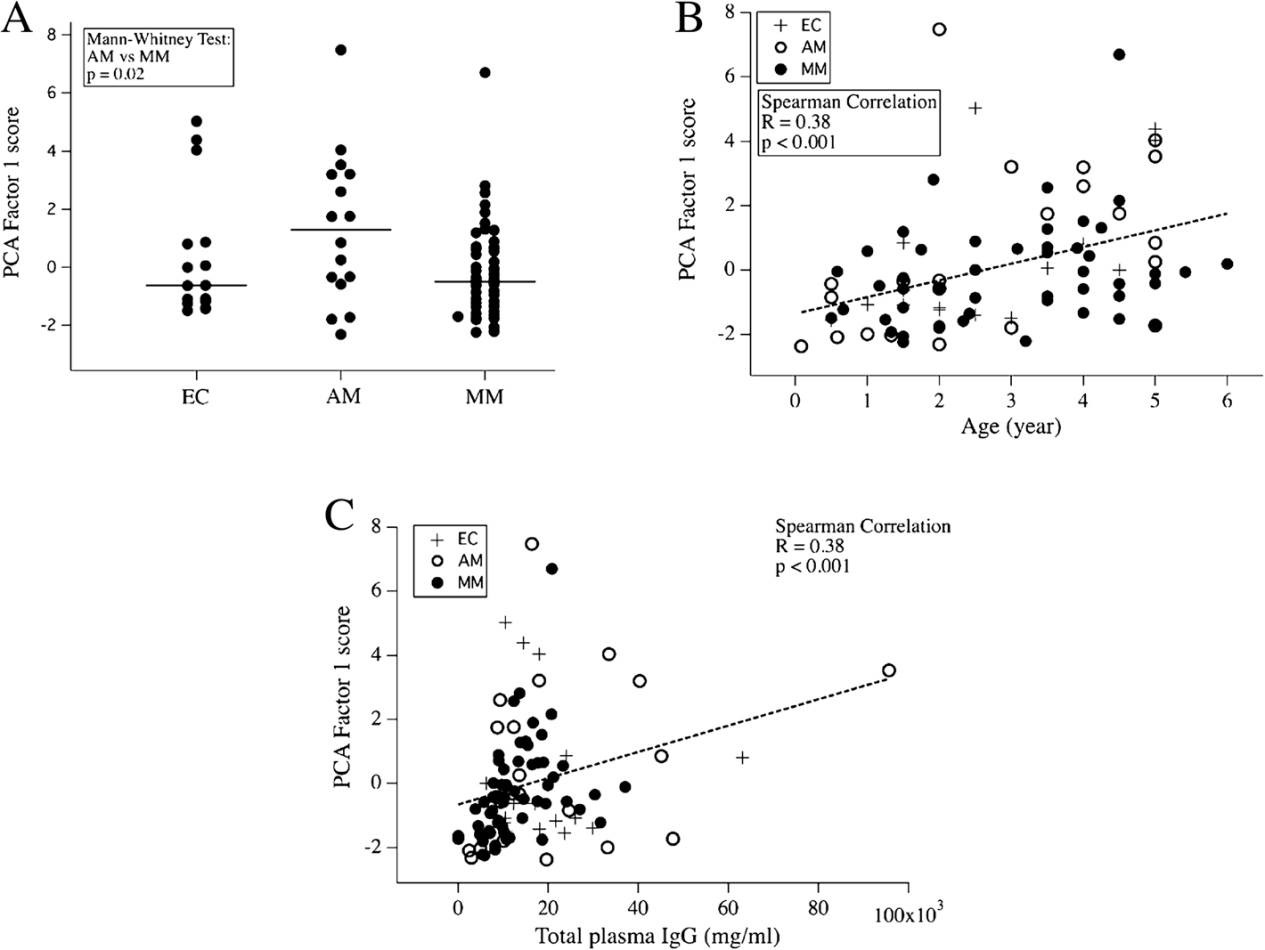
Patterns of brain self-reactive IgG responses in EC, AM and MM groups. PCA-1 scores for **(A)** the different groups, **(B)** relation to age, and **(C)** relation to total plasma IgGs.

The self-reactive response induced by *P. falciparum* infection was investigated by measuring auto-antibodies against against brain protein extract using a quantitative immunoblots. Differential repertoire analysis was done on all groups using Principal Component Analysis (PCA). PCA-1 represents a score of total anti-brain activity. Both in non-adjusted and adjusted assays, PCA-1 was higher in the AM group than in MM and EC groups, particularly in patients older than 18 months in whom the presence of maternal IgG could be excluded (Figure 3A). Anti-brain reactivity (PCA-1 score) was also significantly correlated with age (Spearman R = +0.45, p <0.001) (Figure 3B) and total plasma IgG concentration (Spearman R = +0.38, p <0.001) (Figure 3C) in all groups. However, there was no relationship between the reactivity profile and sex of the individual or parasitaemia. The overall number of brain antigens (bands) recognized by plasma IgG was not different among the three groups. Single linkage hierarchical clustering allowed to detect two clusters of self-antigens (section 0–5, corresponding to high molecular weight antigens, and section 25–29, with relatively low molecular weight which were recognized by almost all individuals in the study (see Additional file 3). However, because of the high variability, no particular signature of reactivity could be identified in AM and MM patients, either by clustering or by fold change comparison (see Additional file 4).

### Relationship between self-reactive and *Plasmodium falciparum*-specific antibody responses

**Figure 4:**
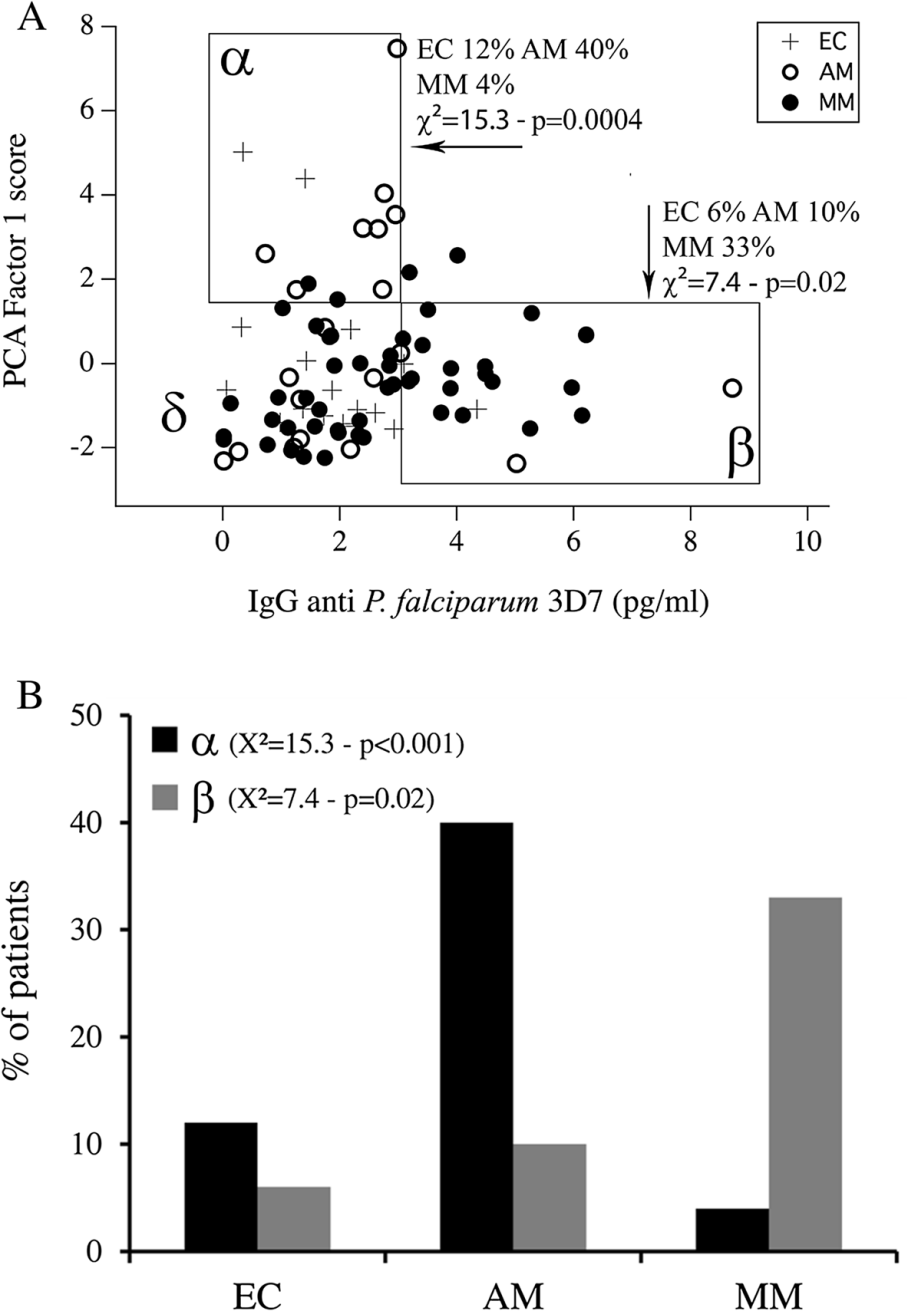
Relationship between auto-antibody response and *Plasmodium falciparum*-specific antibody response. **(A)** Relationship of the auto-antibody response to anti-P. *falciparum* IgG response in the EC, AM and MM groups, and division of response patterns into three sub-groups: α (anti-*P. falciparum* IgG <3 pg/ml; F1 > 1.5), β (anti-*falciparum* IgG >3 pg/ml; F1 < 1.5), δ anti-*falciparum* IgG <3 pg/ml; F1 < 1.5). **(B)** Frequencies of patients in α and β sub-groups in the EC, AM and MM groups.

The subjects were grouped according to anti-brain (PCA-1 score > +1.5) and anti-*P. falciparum* (>3 pg/mL) IgG reactivity. With the exception of two, all study participants fell into three sub-groups: α) high self-reactive IgG and low *P. falciparum*-specific IgG; β) low self-reactive IgG and high *P. falciparum*-specific IgG; and, δ) low self-reactive IgG and low *P. falciparum*-specific IgG (Figure 4A). Participants were unequally distributed between sub-groups. AM individuals were over-represented in sub-group α (40 AM *versus* 12% EC and 4% MM; *χ*2 = 15.3; p = 0.0004) (Figure 4B). Conversely, sub-group β contained more MM (33% MM *versus* 10% AM and 6% EC, *χ*2 = 7.4; p = 0.02) (Figure 4B). Most EC children were classified into sub-group δ. These data clearly suggest that AM is associated with a predominant self-antibody response against brain antigens and low parasite-specific IgG while MM is associated with low self-reactive antibodies but high parasite-specific response.

### Cytokines associated with *Plasmodium falciparum*-specific and self-reactive antibody responses in AM and MM groups

**Figure 5:**
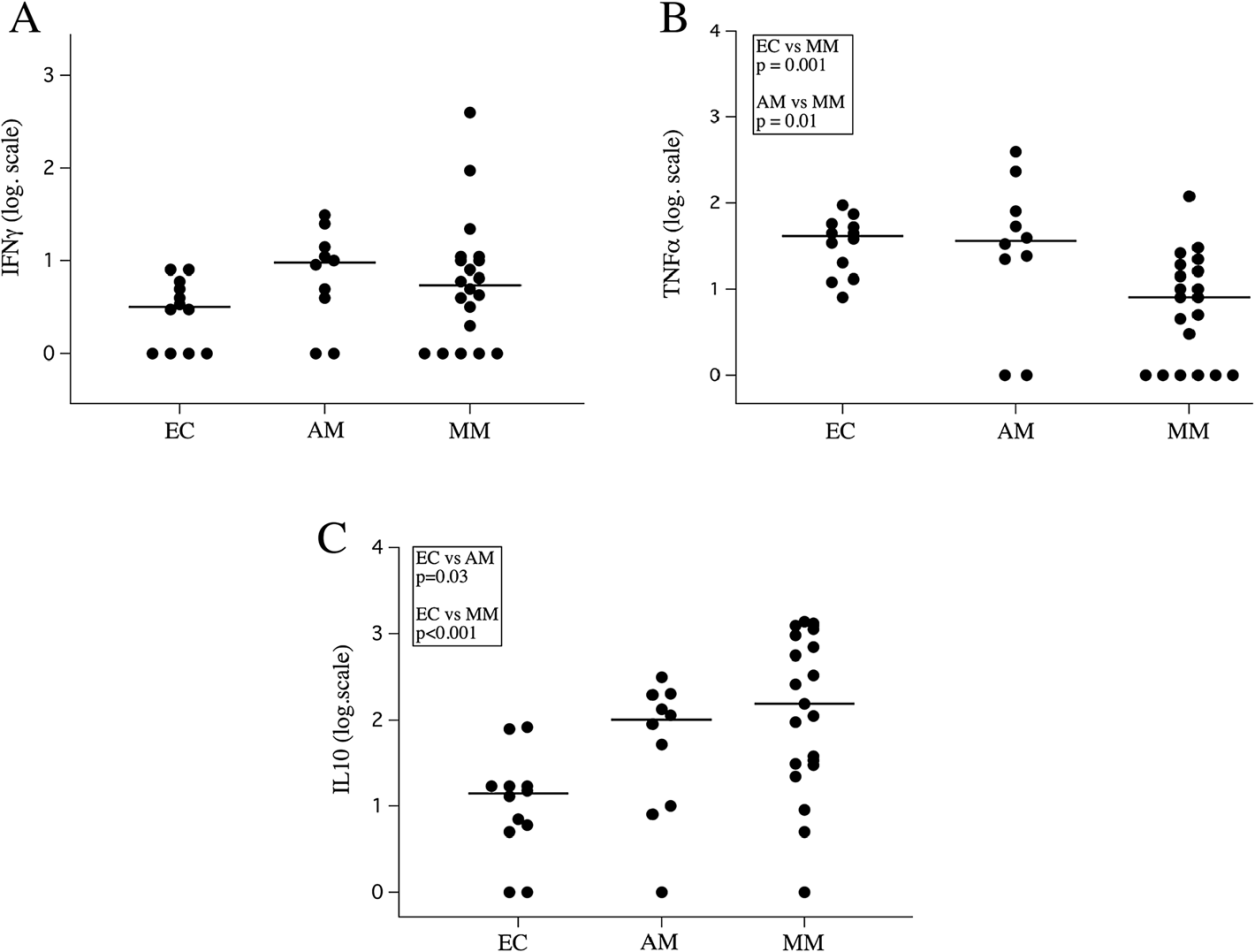
Cytokines levels in respect to clinical status. Plasma levels of IFN-γ **(A)** TNF **(B)** and IL-10 **(C)** in the EC, AM and MM groups at the day of hospitalization and before treatment.

Levels in plasma of IFN-γ, TNF and IL-10 were assessed in all children. Plasma concentrations of IFN-g-γ were similar in the three groups (Figure 5A), whereas TNF levels were significantly lower in MM than EC or AM (p = 0.001 and 0.01, respectively) (Figure 5B). In contrast, IL-10 levels were significantly increased during infection in AM and MM groups (p = 0.03 and p < 0.001, respectively; Figure 5C). IL-10 concentrations was significantly correlated with parasite load on day 0 in infected children (R-Spearman = +0.71; p < 0.001) (Figure 5D) and decreased significantly at day 7 in MM patients after treatment with anti-malarial drugs (p = 0.007, paired *t*-test), when no parasites were detectable in peripheral blood (see Additional file 5). However, there was no correlation between cytokine responses and age.

**Figure 6:**
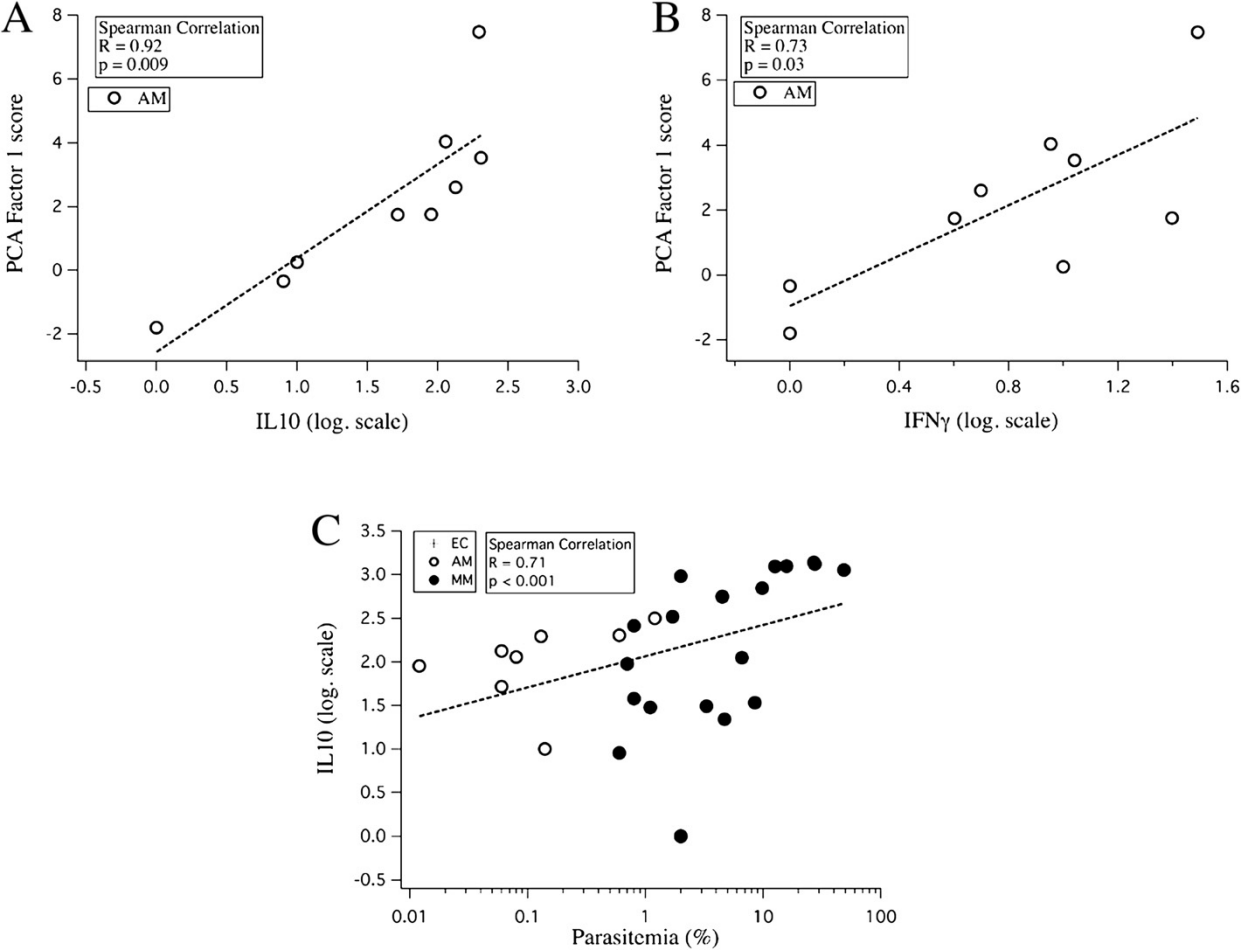
Interaction between cytokine response and auto-antibody patterns. Correlation between self-reactivity (PCA-1 score) and **(A)** IL-10 plasma levels or **(B)** IFN-g plasma levels in AM patients older than 18 months. **(C)** Correlation between IL-10 plasma concentrations and parasite loads at the day of hospitalization and before treatment.

To get an insight into networks involved in protective mechanisms against clinical disease in asymptomatic carriers, the association between cytokine profiles with self-reactive and/or anti-*P. falciparum* IgG responses were analysed using spearman correlation*.* Interestingly, IgG to brain proteins were significantly correlated with IL-10 levels in AM children over 18 months (Spearman R = +0.92, p = 0.009) (Figure 6A). The same trend was also observed for IFN-γ (Spearman R = +0.73, p = 0.03) (Figure 6B). In addition, IL-10 concentrations significantly correlated with parasite load on day 0 especially in *P. falciparum* infected patients (R-Spearman = +0.71; p < 0.001) (Figure 6C).

## Discussion

An infinitesimally small fraction of antibodies produced during *P. falciparum* infection recognize parasite antigens; the rest are against unrelated or host antigens [10,19]. To get an insight into the role played by these auto-antibody in asymptomatic malaria, the relationship between parasite-specific antibody responses and the self-reactive repertoire was investigated in children with uncomplicated malaria and in controls from an endemic area of Libreville, Gabon. In addition, the plasma cytokine profiles were also examined to determine possible associations between such profiles and antibody responses. Unlike previous studies that assessed the protective role of auto-antibodies in malaria using a few antigens, the self-reactivity repertoire in the three groups of patients was evaluated using an approach that allowed the analysis of a large number of antigens [16,17].

Surprisingly, the asymptomatic carriers displayed greater self-reactive IgG response than MM children. The auto-antibody response was correlated with age and total IgG levels, suggesting a link with premunition [2,11,12]. These findings corroborate previous studies demonstrating a high IgG self-reactivity to brain antigens in asymptomatic *P. falciparum* and *Plasmodium vivax* patients [16]. However, levels of *P. falciparum-*specific antibodies were lower in AM than in MM. Antibody responses to MSP1-19 and MSP1-B2 PAR antigens were predominant in MM, whereas AM was associated with low levels of antibody to MSP1-19 but higher levels of antibodies to MSP3 and MSP2. These observations suggest that parasite control does not depend upon anti-*P. falciparum* antibody levels but is linked to the nature and effector functions of those antibodies – such as cytophilic antibodies – that contribute to parasite elimination [13-22]. Antigens expressed by merozoites during blood-stage infection could be targets of these protective antibodies. Indeed, the IgG1 and IgG3 sub-classes have been shown to play a role in anti-merozoite responses [23,24]. Children in areas of high *P. falciparum* transmission are known to develop a strong antibody response to PfEMP1 [25,26]. However, in this study, the anti-PfEMP1 antibody response was weak and did not correlate with reduced risk of clinical disease. This discrepancy could result from genetic differences among the studied populations.

A correlation between elevated self-reactivity to brain antigens and plasma levels of IFN-γ and IL-10 was observed in the AM group. IL-10 is an anti-inflammatory cytokine with a controversial role in malaria; high concentrations of IL-10 are associated with both severe disease and protection [18,27]. In addition, plasmatic levels of IL-10 correlated positively with parasite load and decreased dramatically within a week of amodiaquine treatment, concomitant to the disappearance of the parasite from peripheral blood as reported [28,29]. Together, these observations strongly suggest that in AM children, clinical protection to malaria is associated with an increased of self-reactive response, low levels of *P. falciparum-*specific IgG, high plasma levels of IFN-γ and IL-10, while MM was associated with high levels of IL-10 and specific anti-*falciparum* antibodies but low self-reactive response with low TNF secretion.

There was no correlation between parasite-specific and self-reactive antibody responses in the infected groups. However, a self-antigen candidate was detected in the AM group. This suggests that the self-reactive response during *P. falciparum* infection may not be exclusively attributable to cross-reactivity between parasite and host antigens or to polyclonal B cell activation. The origin of the self-reactive antibodies induced during malaria remains unclear. Auto-antibodies associated with AM might be derived from CD27^+^ marginal zone B cells that are expanded in some auto-immune diseases. Alternatively, some of these auto-antibodies could be ‘natural antibodies’ that are produced in the absence of exogenous antigenic stimulation and that are characterized by low affinity for the antigen and polyreactivity [8,10,23]. Auto-antibodies may participate in protective mechanisms by favouring parasite clearance, which could explain the association of a high self-reactive response and with low parasitaemia (<1%) observed in AM [21-24]. Indeed, this has been proposed as a mechanism of haemoglobinopathy-associated protection against malaria [29-36].

## Conclusions

The findings of a strong correlation between autoreactivity and plasma cytokine profile, low *P. falciparum*-specific IgG levels and low parasitaemia in AM children provide new clues into the mechanisms underlying AM. They also strongly suggest a role for the auto-antibody response in protective mechanisms against clinical disease in *P. falciparum-*infected individuals living in endemic areas.

## Additional files

Additional file 1:
**Antibody response to MSP1619 in respect to malaria clinical status.** Description: Plasma levels of IgG recognizing MSP1-19 (pg/mL) antigens in the EC, AM and MM groups at the day of hospitalization and before treatment. Additional file 2:
**Dynamic of the specific antibody response to** ***Plasmodium falciparum.*** Description: Example of dynamic of anti- *P. falciparum* antibodies between day 0 and day 30 in patients n° 13, 120 and 167 from MM; individual n° 50 from EC and n° 64 from AM. Additional file 3:
**Clustering of brain self-reactive IgG responses.** Description: Single linkage hierarchical clustering of the brain self-reactive IgG responses patients of EC, AM and MM groups. EC: Endemic controls, AM: Asymptomatic P. falciparum infected patients, MM: Mild malaria. Additional file 4:
**HeatMap of brain self-reactive IgG responses.** Description: Heat map representing fold change and Mann–Whitney p-value of the brain self-reactive IgG responses comparing each group of patients with the other two groups: MM vs EC, MM vs AM, EC vs AM. Additional file 5:
**Kinetics of** **IL-10** **production in EC, AM and MM groups.** Description: Individual kinetics of IL-10 plasma concentrations between day 0 (before treatment, 7 and day 30 (after treatment) in patients of EC, AM and MM groups. EC: Endemic controls, AM: Asymptomatic *P. falciparum* infected patients, MM: Mild malaria.

## Abbreviations

AM: Asymptomatic malaria
MM: Mild malaria
EC: Endemic controls
AMA: Apical membrane antigen
GST: Glutathione-S-transferase
MBP: Myelin binding protein
MSP: Merozoite surface protein
NANP: Tetrapeptide of *Plasmodium falciparum* circumsporozoite protein
PCA: Principal component analysis
PfEMP: *Plasmodium falciparum* erythrocyte membrane protein
