# Supplementary figures and images for: Asymptomatic Plasmodium falciparum infection in children is associated with increased auto-antibody production, high IL-10 plasma levels and antibodies to merozoite surface protein 3
Source: Malar J. 2015 Apr 16;14:162. doi: 10.1186/s12936-015-0658-7 (PMC4419484; doi:10.1186/s12936-015-0658-7)

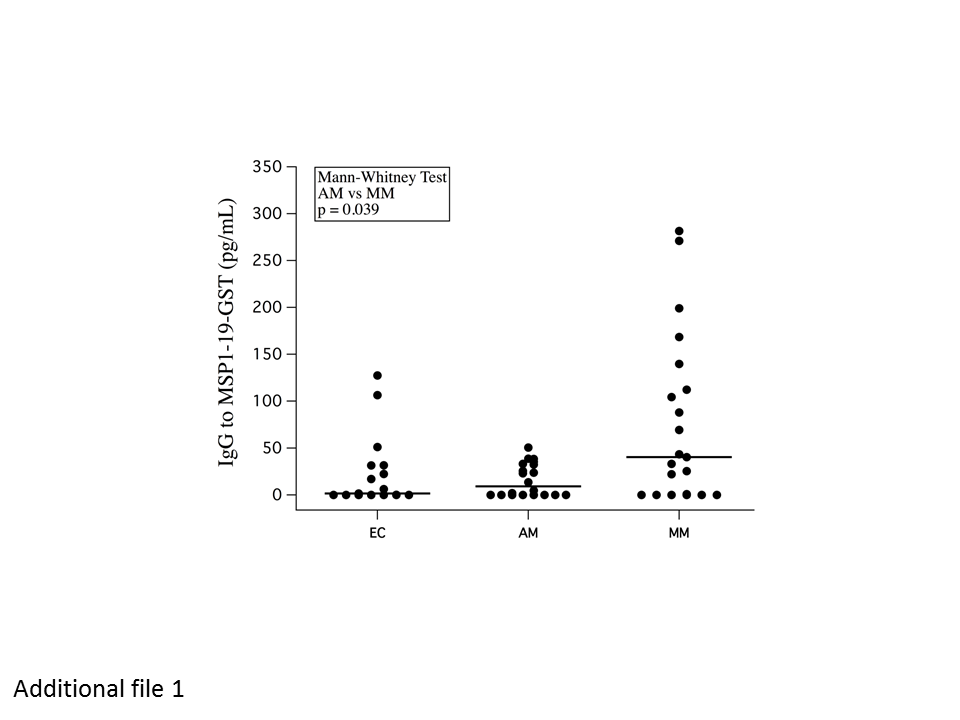

Supplement: Additional file 1: — Antibody response to MSP1619 in respect to malaria clinical status. Description: Plasma levels of IgG recognizing MSP1-19 (pg/mL) antigens in the EC, AM and MM groups at the day of hospitalization and before treatment. [file 12936_2015_658_MOESM1_ESM.png]

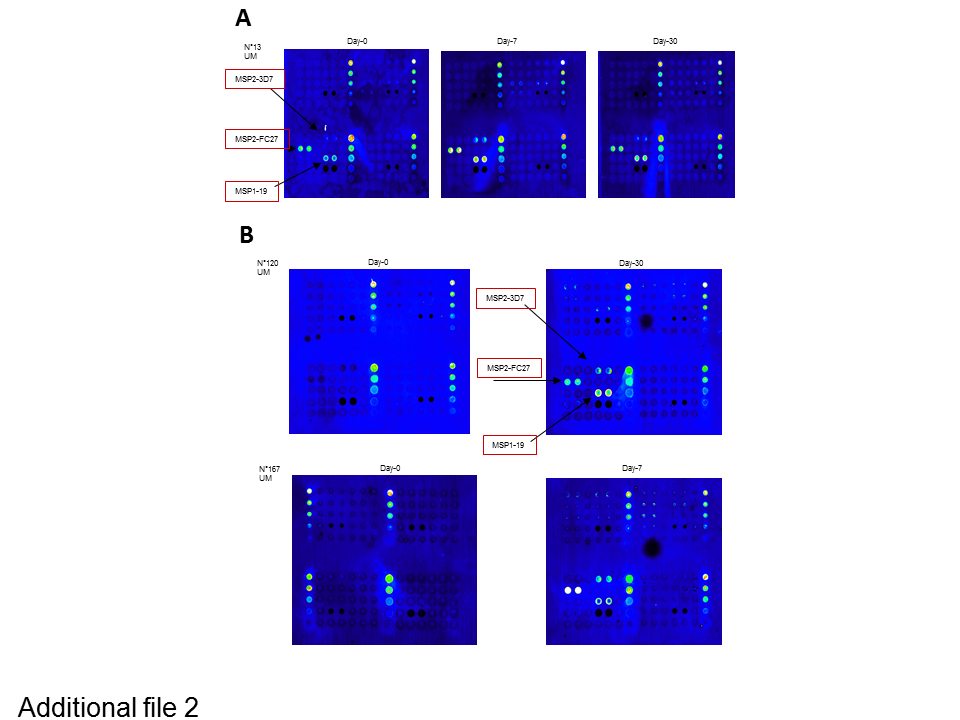

Supplement: Additional file 2: — Dynamic of the specific antibody response to Plasmodium falciparum. Description: Example of dynamic of anti- P. falciparum antibodies between day 0 and day 30 in patients n° 13, 120 and 167 from MM; individual n° 50 from EC and n° 64 from AM. [file 12936_2015_658_MOESM2_ESM.zip › 12936_2015_658_add2A,B.png]

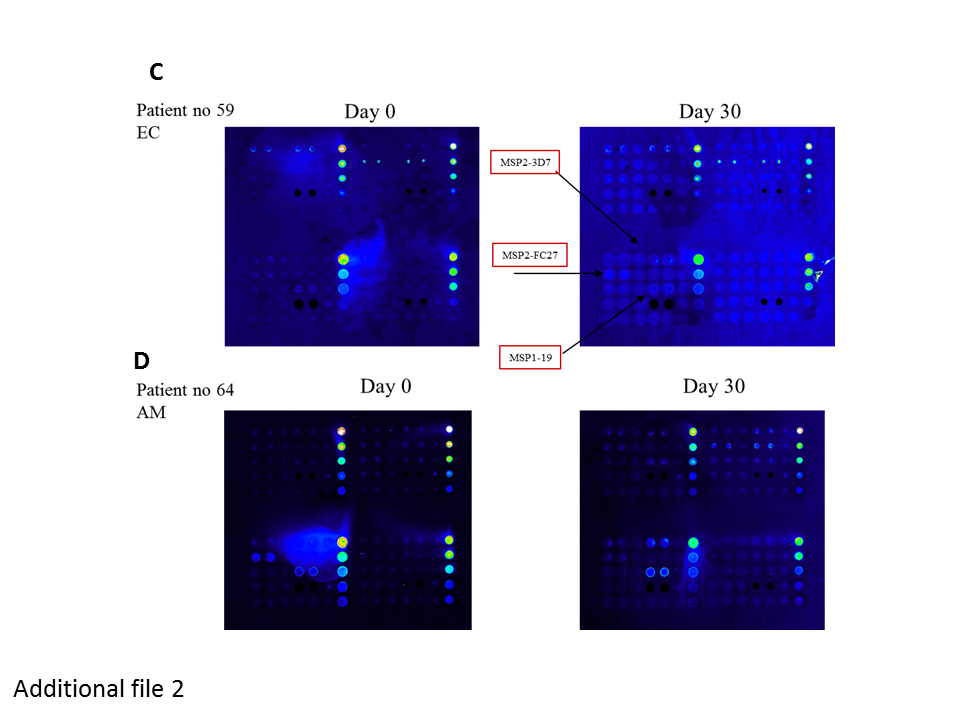

Supplement: Additional file 2: — Dynamic of the specific antibody response to Plasmodium falciparum. Description: Example of dynamic of anti- P. falciparum antibodies between day 0 and day 30 in patients n° 13, 120 and 167 from MM; individual n° 50 from EC and n° 64 from AM. [file 12936_2015_658_MOESM2_ESM.zip › 12936_2015_658_add2C,D.png]

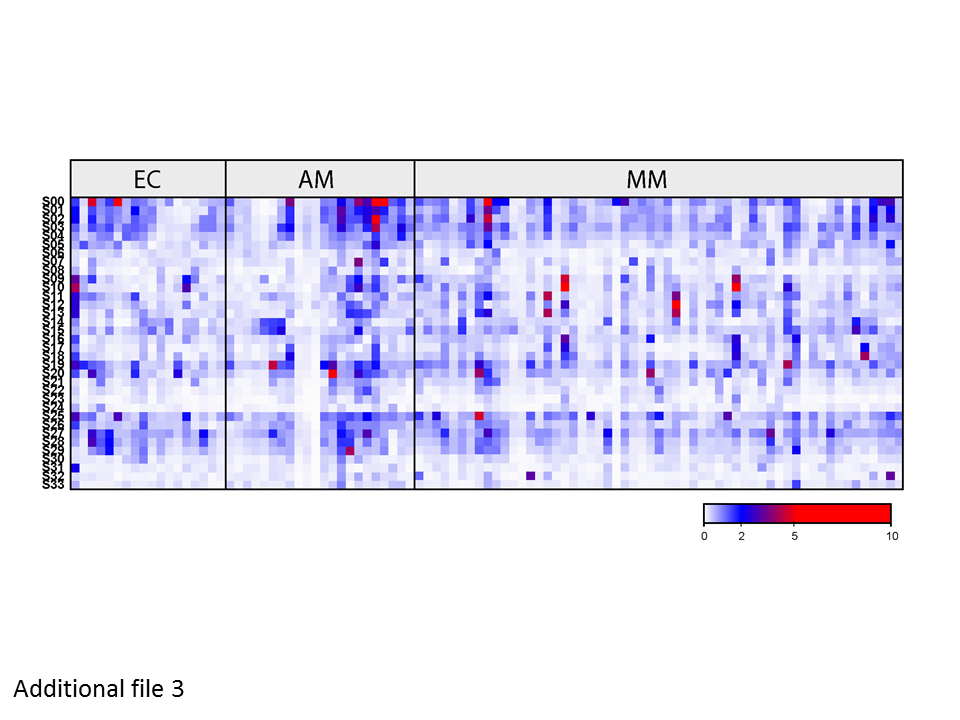

Supplement: Additional file 3: — Clustering of brain self-reactive IgG responses. Description: Single linkage hierarchical clustering of the brain self-reactive IgG responses patients of EC, AM and MM groups. EC: Endemic controls, AM: Asymptomatic P. falciparum infected patients, MM: Mild malaria. [file 12936_2015_658_MOESM3_ESM.png]

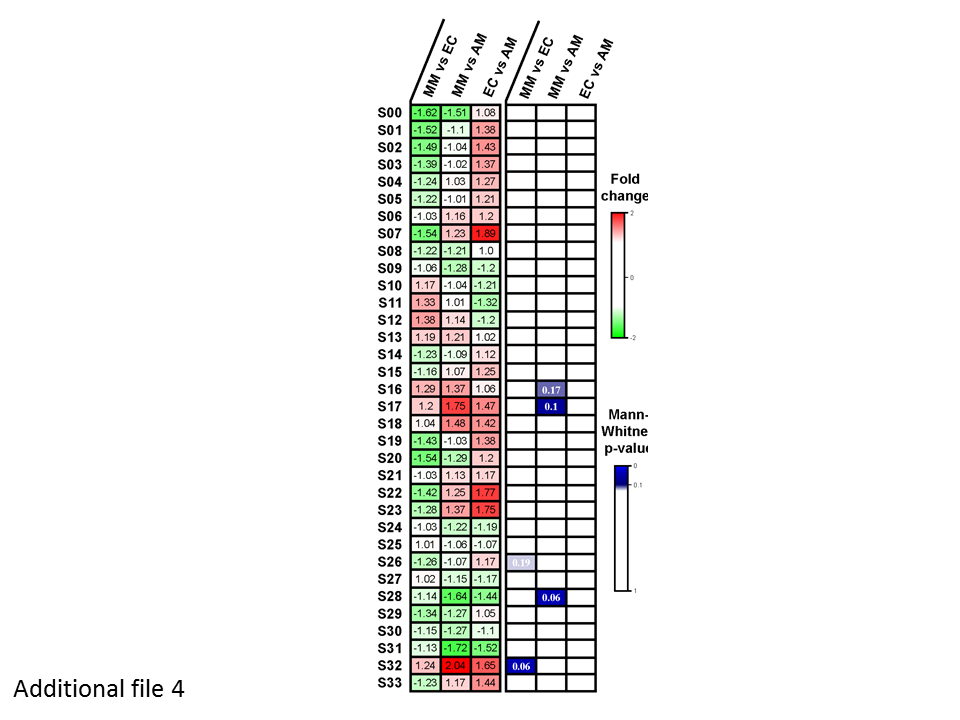

Supplement: Additional file 4: — HeatMap of brain self-reactive IgG responses. Description: Heat map representing fold change and Mann–Whitney p-value of the brain self-reactive IgG responses comparing each group of patients with the other two groups: MM vs EC, MM vs AM, EC vs AM. [file 12936_2015_658_MOESM4_ESM.png]

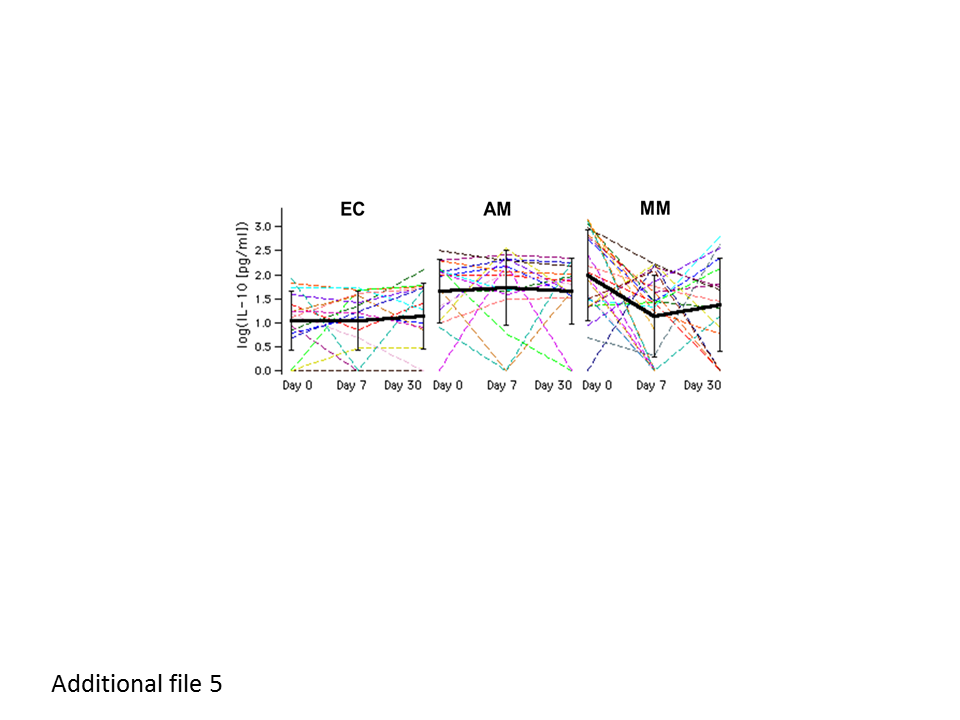

Supplement: Additional file 5: — Kinetics of IL-10 production in EC, AM and MM groups. Description: Individual kinetics of IL-10 plasma concentrations between day 0 (before treatment, 7 and day 30 (after treatment) in patients of EC, AM and MM groups. EC: Endemic controls, AM: Asymptomatic P. falciparum infected patients, MM: Mild malaria. [file 12936_2015_658_MOESM5_ESM.png]
